# A Key Role for NF-**κ**B Transcription Factor c-Rel in T-Lymphocyte-Differentiation and Effector Functions

**DOI:** 10.1155/2012/239368

**Published:** 2012-03-06

**Authors:** Alexander Visekruna, Anton Volkov, Ulrich Steinhoff

**Affiliations:** ^1^Institute for Medical Microbiology and Hygiene, Philipps University of Marburg, Hans Meerwein Straße 2, 35032 Marburg, Germany; ^2^Department of Immunology, Max Planck Institute of Infection Biology, 10117 Berlin, Germany

## Abstract

The transcription factors of the Rel/NF-**κ**B family function as key regulators of innate and adoptive immunity. Tightly and temporally controlled activation of NF-**κ**B-signalling pathways ensures prevention of harmful immune cell dysregulation, whereas a loss of control leads to pathological conditions such as severe inflammation, autoimmune disease, and inflammation-associated oncogenesis. Five family members have been identified in mammals: RelA (p65), c-Rel, RelB, and the precursor proteins NF-**κ**B1 (p105) and NF-**κ**B2 (p100), that are processed into p50 and p52, respectively. While RelA-containing dimers are present in most cell types, c-Rel complexes are predominately found in cells of hematopoietic origin. In T-cell lymphocytes, certain genes essential for immune function such as *Il2* and *Foxp3* are directly regulated by c-Rel. Additionally, c-Rel-dependent IL-12 and IL-23 transcription by macrophages and dendritic cells is crucial for T-cell differentiation and effector functions. Accordingly, c-Rel expression in T cells and antigen-presenting cells (APCs) controls a delicate balance between tolerance and immunity. This review gives a selective overview on recent progress in understanding of diverse roles of c-Rel in regulating adaptive immunity.

## 1. Introduction

c-Rel is a member of the Rel/NF-*κ*B family of eukaryotic transcription factors, which also includes the proteins RelA (p65), RelB, NF-*κ*B1 (p105/p50), and NF-*κ*B2 (p100/p52). NF-*κ*B transcription factors can form various homo- and heterodimers possessing unique specificities in regulating target gene expression [1]. Despite some redundancy, functional studies on mice lacking one or more NF-*κ*B proteins revealed that distinct NF-*κ*B subunits play specific role in regulating T-cell development and effector functions [2–5]. NF-*κ*B complexes are held in the cytoplasm by interacting with a family of inhibitory proteins known as the I*κ*B proteins. In general, binding of I*κ*B proteins to NF-*κ*B dimers masks the nuclear localization signals of NF-*κ*B proteins and inhibits both, nuclear import of NF-*κ*B complexes as well as binding to their specific DNA binding site (*κ*B site) [6]. Activation of NF-*κ*B complexes in T lymphocytes requires T-cell receptor (TCR) stimulation, which provides a signal for phosphorylation and degradation of I*κ*B proteins via the ubiquitin-proteasome system in order to initiate nuclear translocation and DNA binding of active NF-*κ*B dimers [7]. In the past decade, several studies have been conducted to identify genes that are directly regulated by the transcription factor c-Rel [8–12]. Despite extensive research, not all c-Rel-controlled genes have been identified yet. Most information about the role of c-Rel in T lymphocytes has come from *in vivo* analyses of c-Rel deficient mice suggesting an important function for this protein in regulating T cell development, differentiation, and effector function in thymus and peripheral lymphoid tissues. This review attempts to highlight various nonredundant physiological functions of c-Rel, particularly with regard to regulation of T-cell-mediated immunity.

## 2. c-Rel-Signalling Pathway in T Lymphocytes

**Figure 1:**
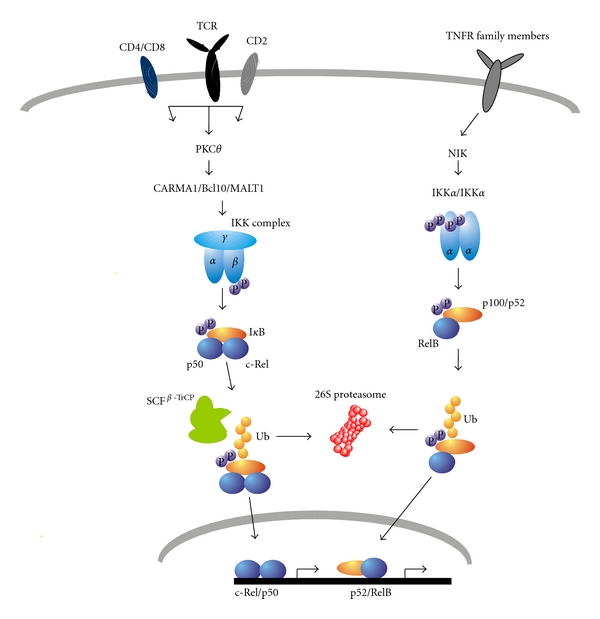
Canonical and alternative NF-*κ*B activation pathways in T cells. Several signals are required for the activation of the canonical NF-*κ*B-signalling pathway. The inhibitory I*κ*B proteins typically bind to dimers of the NF-*κ*B family such as p65/p50 (not shown in the figure) and c-Rel/p50 to generate inactive complexes that are sequestered in the cytosol. PKC*θ* is a central molecule for TCR-mediated NF-*κ*B activation. Phosphorylation of CARMA by PKC*θ* results in formation of stable CARMA/Bcl10/MALT1 complex and activation of IKK. Activated IKK*β* kinase mediates phosphorylation of I*κ*B molecules and recruitment of SCF^*β*-TrCP^ ubiquitin ligase. Activation of alternative NF-*κ*B pathway is triggered by a subset of TNFR family members and is mediated by NIK and IKK*α* that phosphorylates p100. The ubiquitin-proteasome pathway is involved in activation of NF-*κ*B via specific degradation of I*κ*Bs and processing of p100 to produce p52.

Three main NF-*κ*B activating pathways exist in mammalian cells [13]. The so-called canonical NF-*κ*B pathway by which cytokines and other various signals initiate activation of RelA/p50 and c-Rel/p50 heterodimers has been investigated in greatest detail. A central component in NF-*κ*B regulation is a serine-specific I*κ*B kinase (IKK), a complex composed of three subunits: IKK*α* (IKK1), IKK*β* (IKK2), and IKK*γ* (NEMO). In T lymphocytes, the canonical pathway is triggered by TCR and CD28 engagement resulting in activation of IKK*α*/IKK*β*/IKK*γ* complex. Following stimulation, activation of IKK results in phosphorylation of I*κ*Bs on specific serine residues, recruitment of the SCF^*β*-TrCP^ ubiquitin ligase complex, rapid polyubiquitination, and subsequent degradation of I*κ*B inhibitory proteins by the 26S proteasome [14]. I*κ*B*α* is phosphorylated by IKK*β* on two N-terminal serine residues, Ser32 and Ser36, which creates a binding site for the receptor subunit (*β*-TrCP) of specific ubiquitin E3 ligase SCF. Once liberated from I*κ*B molecules, p65/50 and c-Rel/p50 dimers participate in the transcriptional regulation of distinct genes involved in adaptive immunity functions. In contrast, the alternative NF-*κ*B activation pathway is induced by a subset of TNFR family members (e.g., LT*β*R and BAFFR) involving NIK and IKK*α*-mediated p100 processing and generation of transcriptionally active p52/RelB heterodimers [15, 16] (Figure 1). The major function of this pathway is related to the development and organization of secondary lymphoid organs (downstream of LT*β*R) and homeostasis of B cells (downstream of BAFFR) [6, 17]. Although T cells express a number of costimulatory TNFR family members such as OX40, CD30, and GITR that are assumed to induce processing of p100 via activation of NIK and IKK*α* homodimers [18, 19], it remains largely unclear how the alternative NF-*κ*B pathway exactly regulates T-cell differentiation, effector functions, and memory responses. The third NF-*κ*B pathway, also called p105 pathway, is initiated by IKK*β* through phosphorylation of p105 precursor protein at Ser927 and Ser932. It uses the same IKK complex as the canonical pathway and its activation lead to complete degradation of the p105 molecule and release of docked molecules [14, 20, 21].

Since the discovery of NF-*κ*B proteins 25 years ago, there have been many questions with respect to the selectivity and diversity of NF-*κ*B functions. Novel studies have begun to reveal how the complex networks of positive and negative regulatory signals and crosstalk between activating pathways shape the NF-*κ*B response in a cell-type-dependent and stimulus-specific way [17, 22–24]. In naïve T cells, TCR stimulation and subsequent IKK*β*-dependent phosphorylation of I*κ*B*α* lead to the nuclear translocation of active NF-*κ*B dimers. The protein kinase C isozyme PKC-*θ* is a central molecule for recruiting additional factors required for IKK-mediated NF-*κ*B activation in T cells. TCR-mediated activation of p65/p50 and c-Rel/p50 dimers in T cells includes activation of kinases of the Src and the Syk families. Furthermore, CD28 and TCR costimulation facilitates phosphorylation of CARMA1 and its recruitment into signalling complex with Bcl10 and MALT1 (CBM complex, Figure 1). Although not completely elucidated, mechanisms such as linear ubiquitination of NEMO and phosphorylation of IKK*β* (probably by TAK1) lead to the activation of the IKK complex and phosphorylation of I*κ*Bs [16]. Cellular localization of NF-*κ*B proteins is controlled by three I*κ*B isoforms: I*κ*B*α*, I*κ*B*β* and I*κ*B*ε*. Interestingly, the rate of degradation and resynthesis of each I*κ*B isoform may vary in cell-specific way [25]. Whereas I*κ*B*α* mediates rapid NF-*κ*B activation and strong negative feedback loop regulation, I*κ*B*β* and I*κ*B*ε* allow a relatively stable NF-*κ*B response by responding more slowly and acting to dampen oscillatory NF-*κ*B activation profile [25–27]. An important question is how the closely related RelA and c-Rel proteins can operate distinctly in T lymphocytes and whether the inhibitory I*κ*Bs play a central role in these processes. Recent studies suggest that triggering the TCR/CD3 complex results in rapid translocation of active p65-containing dimers into the nucleus and slower activation of c-Rel complexes. As consequence, c-Rel-dependent gene transcription in T cells is slower as compared to p65-mediated responses. In unstimulated T cells, c-Rel is primarily associated with I*κ*B*β*, and the proportion of c-Rel bound to I*κ*B*α* can be substantially increased after activation of cells with TNF-*α* and IL-1*β* [28, 29]. In particular, I*κ*B*α* is degraded more rapidly than I*κ*B*β* and I*κ*B*ε*. Taken together, in naïve T cells, two members of the classical NF-*κ*B activating pathway, p65 and c-Rel, seem to be differentially regulated by forming distinct complexes with I*κ*Bs. c-Rel dimers cannot be easily activated as c-Rel is mainly complexed to I*κ*B*β*. Costimulatory signals transmitted by CD80/86/CD28 and the presence of proinflammatory cytokines secreted by APCs increase I*κ*B*β* degradation and c-Rel is consequently shifted to I*κ*B*α*-associated complexes [29].

Remarkably, turnover of c-Rel itself seems to be regulated by the ubiquitin-proteasome pathway adding another level of the complexity to its regulation [30]. A novel study has described that the E3 ubiquitin ligase Peli1 mediates polyubiquitination of c-Rel and subsequent degradation of this protein by the 26S proteasome. This prevents aberrant accumulation of c-Rel during T-cell activation. Interestingly, Peli1 deficiency in mice results in nuclear accumulation of c-Rel, T-cell hyperactivation, and spontaneous development of autoimmunity associated with multiorgan inflammation and production of autoantibodies [31]. This finding emphasizes that regulation of c-Rel expression in T cells might play an important role in the maintenance of peripheral T-cell tolerance.

## 3. Cell-Autonomous Role of c-Rel in T-Lymphocyte Differentiation

The differentiation of the CD4^+^ T-cell lineage into T effector cells is a crucial prerequisite for a successful host immune defense against pathogens. Functional specialization is coordinated by a complex genetic network, initiated and terminated in a time-dependent manner. Several studies have attempted to identify transcriptional signatures and master transcription factors driving the differentiation of individual T-cell subsets. Recently, a discovery of huge range of the functional plasticity and heterogeneity of T-cells has drawn much attention [32, 33]. As several subpopulations have only been examined *in vitro*, it is still unclear if they should be considered as distinct T cell subsets or whether expression of characteristic molecules is just an adaptation of already known and well-described Th1, Th2, and Th17 cells to certain microenvironment. This review aims to discuss the data that will allow us to understand how c-Rel influences the development and effector functions of most important T-cell subsets. Th1 and Th2 cells have distinct immunological functions by producing their key cytokines IFN-*γ* and IL-4, respectively. Recently, IL-17-producing cells that express transcription factors IRF-4 and ROR*γ*t, named Th17 cells, have been described to develop via a unique lineage, independently of the Th1 and Th2 master transcription factors T-bet and GATA-3 [34–37]. Another population of CD4^+^ T cells, so-called follicular helper T cells (T_FH_), preferentially reside in germinal centres where they help B cells to generate high-affine antibodies [38, 39]. Finally, regulatory T (Treg) cells are characterized by their expression of transcription factor Foxp3 and are essential for tolerance and prevention of autoimmunity [40, 41].

The transcription factor c-Rel has emerged to be an important molecule that can mediate proliferation, differentiation, and cytokine production of T cells. However, the extent and impact of the described defects in c-Rel-deficient T-cells vary considerably. Experiments with c-Rel-deficient mice have revealed that this protein is crucial for optimal IL-2 production and expression of IL-2R*α* (CD25) in T cells [42, 43]. Normally, immature T cells are unable to produce IL-2. However, once dendritic cells (DCs) encounter danger signals at the site of infection and get fully maturated, differentiation, of naïve CD4^+^ T cells is driven effectively through antigen recognition, cytokine milieu, and costimulation by CD80 and CD86. In response to antigens, T cells start producing IL-2 and IL-2/IL2-R-signalling becomes crucial for their activation and expansion. In light of the finding that c-Rel complexes are mainly bound to I*κ*B*β* and that stimulation via CD28 leads to degradation of I*κ*B*β* and activation of c-Rel signalling pathway, it is evident why c-Rel-deficient T cells cannot respond appropriately to T-cell activation signals. With regard to activated naïve T cells, c-Rel signalling (acting downstream of TCR and CD28) may also be essential for secretion of other IL-2-dependent cytokines. IL-2 is known to be required for optimal IL-4 and IFN-*γ* expression by T-helper cells and for expression of granzyme and perforin by cytotoxic T lymphocytes (CTL) [44, 45]. Since c-Rel, AP-1, and NFAT act in concert to regulate IL-2 expression and T-cell proliferation, IL-2 secretion is reduced but not completely abrogated in c-Rel-deficient T cells. Thus, some defects in Th differentiation observed under *in vitro* polarizing conditions in the absence of c-Rel may indirectly result from decreased T-cell proliferation. Interestingly, in mature effector T cells that differ from naïve ones by producing cytokines more rapidly after TCR stimulation, IL-2 and IFN-*γ* gene expression seems to occur independently of c-Rel-mediated signal transduction [29].

It is likely that regulatory functions of c-Rel on target gene promotors are accomplished by heterodimerization with p50 or by forming c-Rel/c-Rel homodimers. There is also evidence that c-Rel/p50 dimers cooperate with other NF-*κ*B family members. For example, c-Rel and p65 complexes bind together to IL-2R*α* promoter and even cooperate with other transcription factors such as SRF to increase expression of IL-2R*α* gene [46]. Recently, a c-Rel binding site was identified in proximal promoter of *Il21* gene implicating an important role for c-Rel in development of IL-21-dependent T and B subsets [47]. IL-21 has been reported to be essential for both T_FH_ development and regulation of B-cell function [38, 48–51]. Accordingly, the frequencies of T_FH_ cells and germinal centre (GC) B cells were significantly reduced in c-Rel-deficient mice immunized with MOG_35–55_ [47]. We have also found reduced IL-21 production and GC formation in Peyer's patches of c-Rel-deficient mice (A. Visekruna, unpublished data). However, our recent unpublished results show that, at least in response to IL-6 stimulation, there was no significant difference between WT- and c-Rel-deficient CD4^+^ T cells with respect to IL-21 production. This suggests that c-Rel might be involved in IL-6-independent signal transduction pathways leading to induction of IL-21 expression. Although c-Rel binds to the promoter of the *Il21* gene, many other transcriptional activators such as STAT-3, IRF-4, and NFATc2 seem to be more important for optimal *Il21* gene expression [52, 53].

More recently, c-Rel has been shown to control the differentiation of Treg cells in the thymus by promoting formation of so-called *Foxp3*-specific “enhanceosome [*sic*]” containing p65, Smad3, NFATc2, and CREB [54–58]. It has also become evident that c-Rel protein and RNA expression are specifically upregulated in CD4^+^CD25^+^ thymocytes as compared to other T-cell populations in the thymus indicating the importance of this factor for development and maintenance of emerging Treg population. Intriguingly, although c-Rel-deficient mice exhibit diminished Treg cell numbers, c-Rel appears to be dispensable for immune suppressive activity of Treg cells, as c-Rel-deficient Treg cells are able to inhibit T-cell proliferation *in vitro* and suppress development of T-cell-induced colitis [58]. Three highly conserved noncoding DNA sequences (CNSs) in the *Foxp3* locus have been identified and named CNS1-3. *In silico* analysis has revealed that c-Rel complexes but not p65 complexes bind to CNS3 region of *Foxp3* locus resembling the CD28 response element (CD28RE) in the *Il2* locus, also known to be occupied by c-Rel homodimers [59]. Given the importance of Foxp3 expression in Treg differentiation and effector functions, an interesting consideration point is to better understand how intracellular signalling molecules and adapters are involved in NF-*κ*B activation in Treg population. Although engagement of TCR and IL-2 signalling is crucial for both thymic and peripheral development of Treg cells, the overall “quality” of peripheral signals may not mimic all facets of Treg development in thymus. While TCR signalling via c-Rel provides an instructive signal to open the *Foxp3* locus during thymic development, additional factors are probably involved in the generation of peripheral Treg (iTreg) cells. Very recently, we have demonstrated that, in the presence of TGF-*β*, the addition of exogenous IL-2 is sufficient to drive iTreg differentiation and to upregulate Foxp3 expression in c-Rel-deficient naïve CD4^+^ T cells [60]. Further, our unpublished data suggest that *in vivo* treatment with immune complexes consisting of IL-2 and anti-IL-2 mAb (JES6-1) leads to a widespread increase in Treg cell frequencies not only in WT but also in c-Rel deficient mice. The paradoxical observation that frequencies of Treg cells increase substantially in c-Rel deficient mice implies that, at least in the periphery, control of the *Foxp3 *locus by c-Rel is not required for maintaining the homeostasis and expansion of Treg cells. Interestingly, thymic and peripheral CD4^+^Foxp3^+^ Treg cell frequencies are also significantly reduced in mice deficient in upstream components of c-Rel-activating pathway such as PKC-*θ*, CARMA1, Bcl10, and MALT1 [56, 61–63]. It will be of interest to determine if iTreg cells generated from these mice induce Foxp3 after *in vitro* exposure to IL-2 and TGF-*β* similarly to c-Rel-deficient T cells. These findings collectively suggest that c-Rel has an important nonredundant function for Treg cells by inducing Foxp3 expression during thymic Treg cell development.

Additionally to its role in several CD4^+^ T subsets, c-Rel might play an important role for CD8^+^ T-cell function. One mechanism in particular is regulation of IL-2 production as consumption of this cytokine has a crucial influence on various aspects of CD8^+^ T-mediated immunity. Current experimental data indicate that the PKC-*θ*/c-Rel-signalling axis is a crucial survival pathway in activated CD8^+^ T lymphocytes. Interestingly, exogenous IL-2 can bypass survival and proliferative defects in PKC-*θ*- and c-Rel-deficient CD8^+^ T cells [64]. Additionally, in the presence of exogenous IL2, c-Rel-deficient CTL have normal cytotoxicity *in vitro*. *In vivo* studies have shown normal capacity of c-Rel-deficient CD8^+^ T cells to clear influenza infection [65]. Major contribution of c-Rel to functional CTL responses might comprise regulation of the inflammatory environment (e.g., regulation of cytokines produced by APC and CD4^+^ T cells) rather than playing substantial intrinsic role in cytotoxic T cells.

## 4. Crucial Role of c-Rel in Regulating Inflammation and Immune Defense against Microbial Pathogens

*In vitro *analyses of c-Rel-deficient cells have revealed selective requirement for c-Rel during IL-12 p40 induction in macrophages [66]. Similarly, p50/c-Rel dimers have been described to bind to the proximal promoter of IL-12 p35 and IL-23 p19 subunits in murine macrophages and DC [67–70]. Both proteins, IL-12 and IL-23, play a crucial role for the differentiation of T lymphocytes and immunity against pathogens. Importantly, maturation of DC is not affected in the absence of c-Rel, whereas the loss of this protein in APC compromises DC-mediated CD4^+^ T-cell activation [71]. Thus, c-Rel appears to be a crucial link between innate immune signals and primary T-cell responses by substantially influencing a delicate balance between Th1, Th17, and Treg cells.

Complex *in vivo* functions of different NF-*κ*B family members following exposure to pathogens remain partially controversial. Infected mice devoid of specific NF-*κ*B proteins display distinct phenotypes probably reflecting the ability of individual members to regulate expression of different sets of target genes associated with innate and adoptive immunity. One of the fundamental immunological challenges is to understand how the immune system can decide what type of immune responses to launch against different classes of pathogens. The capacity of Th1 and Tc1 responses to protect against intracellular pathogens is well known. For example, the control of infection with protozoan parasite *Leishmania major* has been attributed to IL-12-mediated differentiation and expansion of CD4^+^ Th1 cells with subsequent IFN-*γ* secretion, activation of infected macrophages, and NO-mediated killing of parasite. Two studies have shown that mice lacking c-Rel display a high susceptibility to* L. major* infection. The reduced levels of IL-12 p70 in DC as well as defective IFN-*γ* secretion by T cells and NO production by macrophages in both *L. major*-infected MyD88 and c-Rel-deficient mice suggest that the high susceptibility of such animals is dependent on TLR-induced activation of c-Rel-signalling pathway with subsequent development of IL-12-mediated protective Th1 response against *Leishmania* parasites [72–74]. One might assume that this mechanism displays a general dependency of protective Th1 immunity on c-Rel, particularly involving regulation of IL-12 production by this transcription factor in APC. Although the failure of c-Rel deficient mice to control infection with another intracellular parasite* Toxoplasma gondii *was also associated with defective Th1 responses, in contrary to infection with *L. major*, this effect appears to be rather dependent on T-cell-intrinsic expression of c-Rel [75]. Thus, the evidence that c-Rel is essential for the production of IL-12 in response to LPS and* Leishmania*, but dispensable for IL-12 production in response to *Toxoplasma,* suggests that this transcription factor is associated with various complex aspects of regulation of innate and adaptive responses required to control infections [76].

**Figure 2:**
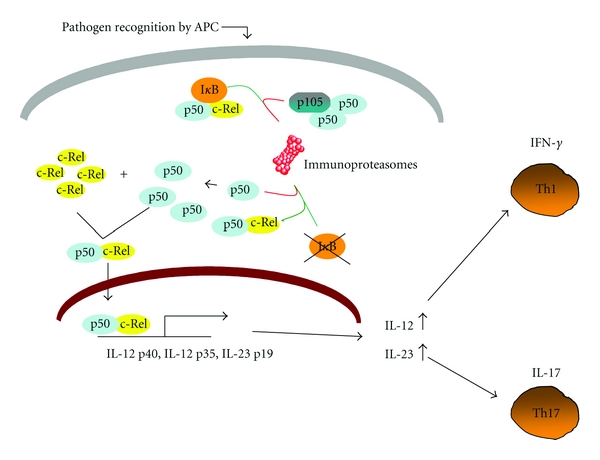
Induction of immunoproteasomes and c-Rel signalling in APC during intestinal inflammation. After disruption of intestinal barrier, the activation of DC by innate immunity triggers such as TLR ligands results in signalling cascade that induces expression of immunoproteasomes. The immunoproteasome is highly active form of proteasome that enhances activation of NF-*κ*B signalling. Additionally, stimulation of TLR releases c-Rel/p50 dimers from I*κ*B to bind to the p40, p35, and p19 promoter. The synergy between immunoproteasomes and c-Rel leads to an increase of IL-12 and IL-23 secretion by APC contributing directly to T-cell-mediated immune responses and exacerbation of intestinal inflammation.

There are emerging insights that c-Rel might play a key role in inflammatory diseases. Recent studies from several groups have shown that c-Rel is essential for the development of both colitis as well as experimental autoimmune encephalomyelitis (EAE). Impaired Th1 and Th17 development seems to occur in parallel with protection from EAE in c-Rel-deficient mice [47, 77, 78]. While potentially multiple roles of c-Rel in the inductive and effector stages of EAE are still partially elusive, its innate function in the control of proinflammatory responses during an intestinal inflammation is well known [79, 80]. A defect in the intestinal epithelial barrier function is an important etiologic factor leading to development of inflammatory bowel disease (IBD) in humans. After encountering microbial agents, activation of c-Rel in DC leads to induction of IL-23 and IL-12 expression. IL-23 strongly enhances production of IL-17 by previously primed CD4^+^ T cells and probably by recently described innate lymphoid cells (ILCs) [81]. The regulation of IL-23 by c-Rel within APC has a critical role in mediating chronic intestinal inflammation. A recent genetic study in humans and several studies in mice have uncovered IL-23 as a key factor in the pathogenesis of Crohn's disease [82–85]. The role of c-Rel and other NF-*κ*B family members can be regulated at many different levels. Two very recent studies have provided important clues to the underlying mechanisms of Th17-cell mediated diseases, showing that c-Rel is required for ROR*γ*t expression in T cells [78, 86]. Therefore, both c-Rel expressed by CD4^+^ T cells regulating directly the expression of a Th17 lineage-specific transcription factor ROR*γ*t as well as c-Rel expression by myeloid cells contribute to differentiation and maintenance of Th17 cells. Results obtained from mouse models and human specimens show that, besides c-Rel-mediated Th17 cell differentiation, IFN-*γ*-mediated induction of immunoproteasomes has an important role for activation of NF-*κ*B and enhancement of chronic inflammation in the gut [80, 87, 88]. Collectively, induction of inflammation in the gut caused by imbalanced activation of DC expressing high level of c-Rel and immunoproteasomes contributes to IBD by augmenting proinflammatory Th1 and Th17 responses (Figure 2). Novel data have also indicated that, in addition to T cells, ILC might be important factors driving intestinal inflammation in mice and humans [89, 90]. However, the role of NF-*κ*B transcription factor c-Rel in regulating various effector functions of these cells has not been characterized yet.

## 5. Conclusions and Future Directions

In last 25 years, major steps forward have been made in understanding how NF-*κ*B regulates different aspects of the immune system. Several studies have begun to examine the role of specific NF-*κ*B family members in regulating infections and chronic inflammatory disorders. c-Rel has emerged to play a critical role in inducing inflammatory and immune responses against pathogens by regulating a crucial set of T-cell stimulatory genes. In addition to dominant effects of c-Rel on promoting Th1- and Th17-mediated immune responses, this transcription factor also plays an important role by providing an initial signal for opening of the *Foxp3* locus in thymic Treg cells. Although the impact of c-Rel on DC activities to induce Th2 responses has not been examined extensively, c-Rel-deficient mice seem to be capable of mounting sufficient Th2 responses. Previous studies suggest that this protein is not essential for control of Th2-mediated intestinal inflammation following *Trichuris muris* infection. In contrast, NF-*κ*B1- and NF-*κ*B2-deficient mice fail to clear helminth infections [91]. Such data reflect nonoverlapping functions of individual NF-*κ*B family members suggesting that targeting specific NF-*κ*B proteins might be a promising therapeutic approach in inflammation and infectious diseases. Especially, molecules that specifically regulate c-Rel-signalling pathway such as E3 ubiquitin ligase Peli1 might be of particular interests as c-Rel exhibits a unique dual capacity to regulate both tolerogenic and inflammatory responses.

## Abbreviations

APC: antigen presenting cell(s)
BAFF: B-cell-activating factor
Bcl10: B-cell lymphoma 10
CREB: cAMP response element-binding protein
DC: dendritic cell(s)
GC: germinal centre
ICAM: intracellular adhesion molecule
IFN: interferon
I*κ*B: inhibitory-*κ*B protein
IKK: I*κ*B kinase
ILC: innate lymphoid cell(s)
IRF: interferon regulatory factor
LT: lymphotoxin
mAb: monoclonal antibody
MALT: mucosa-associated lymphoid tissue lymphoma translocation protein 1
MAPK: mitogen-activated protein kinase
NEMO: NF-*κ*B essential modifier
NFAT: nuclear factor of activated T cells
NF-*κ*B: nuclear factor-*κ*B
NIK: NF-*κ*B inducing kinase
NO: nitric oxide
PKC: protein kinase C
ROR*γ*t: retinoic acid-related orphan receptor *γ*t
Ser: serine
SRF: serum response factor
STAT: signal transducer and activator of transcription
TCR: T-cell receptor
TGF: transforming growth factor
TLR: toll-like receptor.
